# Describing characteristics and treatment patterns of patients hospitalized with COVID-19 by race and ethnicity in a national RWD during the early months of the pandemic

**DOI:** 10.1371/journal.pone.0267815

**Published:** 2022-09-26

**Authors:** Sarah E. Vititoe, Imaani J. Easthausen, Tamar Lasky, Aloka Chakravarty, Marie C. Bradley, Laura M. Roe, Nicolle M. Gatto, Andrew R. Weckstein, Elizabeth M. Garry

**Affiliations:** 1 Scientific Research & Strategy, Aetion, Inc., New York, New York, United States of America; 2 Office of the Commissioner, U.S. Food and Drug Administration, Silver Spring, Maryland, United States of America; 3 Division of Epidemiology, Office of Surveillance and Epidemiology, Center For Drug Evaluation and Research, Food and Drug Administration, Silver Spring, Maryland, United States of America; 4 Columbia Mailman School of Public Health, New York, New York, United States of America; 5 Tulane School of Public Health and Tropical Medicine, New Orleans, Louisiana, United States of America; Istanbul University Istanbul Faculty of Medicine: Istanbul Universitesi Istanbul Tip Fakultesi, TURKEY

## Abstract

**Objective:**

To describe differences by race and ethnicity in treatment patterns among hospitalized COVID-19 patients in the US from March-August 2020.

**Methods:**

Among patients in de-identified Optum electronic health record data hospitalized with COVID-19 (March-August 2020), we estimated odds ratios of receiving COVID-19 treatments of interest (azithromycin, dexamethasone, hydroxychloroquine, remdesivir, and other steroids) at hospital admission, by race and ethnicity, after adjusting for key covariates of interest.

**Results:**

After adjusting for key covariates, Black/African American patients were less likely to receive dexamethasone (adj. OR [95% CI]: 0.83 [0.71, 0.96]) and more likely to receive other steroids corticosteroids (adj. OR [95% CI]: 2.13 [1.90, 2.39]), relative to White patients. Hispanic/Latino patients were less likely to receive dexamethasone than Not Hispanic/Latino patients (adj. OR [95% CI]: 0.69 [0.58, 0.82]).

**Conclusions:**

Our findings suggest that COVID-19 treatments patients received in Optum varied by race and ethnicity after adjustment for other possible explanatory factors. In the face of rapidly evolving treatment landscapes, policies are needed to ensure equitable access to novel and repurposed therapeutics to avoid disparities in care by race and ethnicity.

## Introduction

SARS-COV-2 cases reached 6.1 million in the United States by August 31st, 2020 [1]. Given the novelty of the SARS-COV-2 virus and COVID-19, the first 6 months of the pandemic were characterized by rapid shifts in collective knowledge, clinical care for hospitalized patients, and candidate drugs for COVID-19 treatment [2]. Meanwhile, the COVID-19 pandemic continues to highlight existing racial and ethnic *health disparities*, or differences in the care and/or outcomes of those who have “systematically experienced greater obstacles to health based on their racial or ethnic group” [3]. The burden of the pandemic has disproportionately impacted racial and ethnic minority groups, as evidenced by higher rates of COVID-19 cases, hospitalizations, and deaths [4–8]. Several studies have characterized disparities in COVID-19 cases and outcomes by race and ethnicity using real-world data (RWD); however, only a few describe hospitalized COVID-19 patients [9–11].

To our knowledge, no study characterized inpatient COVID-19 treatment patterns by race and ethnicity while controlling for other possible explanatory factors [12, 13]. We sought to better understand potential racial and ethnic disparities by describing treatments received at admission among COVID-19 patients hospitalized in the U.S. after accounting for baseline comorbidities, disease severity, and predictors of severe disease.

## Methods

This study uses Optum de-identified COVID-19 electronic health records (EHR) data, which include patients with test results for SARS-COV-2 or diagnosis codes associated with COVID-19, sourced from medical and administrative encounters from hospitals, emergency departments, outpatient centers, and laboratories from across the U.S. (N = 2,018,728) **(S3 Table and S3 Fig).** Previous studies have been published using the Optum COVID-19 EHR data [14, 15].

We identified patients hospitalized with a COVID-19 diagnosis during the first six-months of the pandemic in the U.S. (March 1—August 31, 2020). Patients entered the cohort on hospital admission (Day 0), and were required to have either a COVID-19 diagnosis (ICD-10: U07.1) or a SARS-COV-2 diagnostic test with a result of “positive” or “presumed positive” within 21 days prior to admission (Day -21 to Day 0). Patients were excluded if they had missing age or sex data or had no observed medical encounters in the 183 days prior to admission to ensure data observability during the baseline period **(S1 Fig).**

Data on race and ethnicity were sourced from EHR records. Raw values for race included “Asian”, “African American”, “Caucasian”, and “Other/Unknown”; we relabeled “African American” as “Black or African American (Black/AA)” [16]. The “Other/Unknown’’ category in the data did not differentiate patients with missing race information from those with recorded values of “Other” or “Unknown”, therefore these data were considered missing for analytic purposes. American Indian/Alaska Native patients, Native Hawaiian/Pacific Islander patients, and multiracial patients were likely captured in this category. Ethnicity values of “Hispanic” and “Not Hispanic” were relabeled as “Hispanic or Latino (Hispanic/Latino)’’ and “Not Hispanic or Latino (Not Hispanic/Latino)” [16].

From the main study cohort, we identified two sub-cohorts: 1) patients with non-missing race (for race-stratified analyses), and 2) patients with non-missing ethnicity (for ethnicity-stratified analyses). This ensured that patients with a missing race value and a non-missing ethnicity value (or vice versa) contributed to stratifications for which informative data were observed. For secondary analyses, we stratified patients by both race and ethnicity among patients with race recorded as Black/AA, White, or Other/Unknown, and ethnicity recorded as Hispanic/Latino or Not Hispanic/Latino. Note that patients with missing race but non-missing ethnicity were included as a separate category in this analysis since 57.5% of Hispanic/Latino patients were missing race data.

Baseline covariates measured in the 183 days prior to admission included demographic characteristics, indicators of frailty, comorbidities (e.g., Charlson-Quan comorbidity and *high-risk conditions*; (see **Table 1** for a comprehensive list) [17–19]. At admission, symptoms indicative of moderate/severe (dyspnea, hypoxia, or pneumonia), and critical (respiratory failure, shock, or organ dysfunction/failure) COVID-19 were recorded [20]. COVID-19 severity was based on the presence of procedures, diagnoses, or patient vitals indicating presence of or need for invasive mechanical ventilation and/or intubation (IMV), supplemental oxygen/noninvasive ventilation (O2/NIV), or neither IMV nor O2/NIV *(code lists available via pre-print)* [15, 21–23]. Standardized Mean Differences (SMDs) were used to evaluate differences in baseline characteristics by race and ethnicity without statistical hypothesis testing [24]. An SMD > 0.10 or < -0.10 was considered to be a meaningful effect size. See **S1 Fig** for a summary of the covariate assessment windows.

**Table 1:** Baseline and admitting characteristics by race, and by ethnicity.

	Overall	By Race					By Ethnicity		
	Total Cohort N = 19,284	Asian N = 448	Black/AA N = 4,454	White N = 10,845	Asian (SMD*)	Black/AA (SMD*)	Hispanic/ Latino N = 2,732	Not Hispanic/ Latino N = 14,183	(SMD†)
**Age**									
mean (sd)	57.73 (18.23)	56.56 (17.38)	56.12 (17.01)	60.44 (18.07)	**-0.219**	**-0.246**	50.14 (17.90)	59.72 (17.66)	**-0.539**
median [IQR]	60 [46, 72]	58 [44, 70]	58 [45, 69]	63 [49, 75]			51 [37, 63]	62 [49, 73]	
**Sex**									
Female; n (%)	10,205 (52.9%)	239 (53.3%)	2,517 (56.5%)	5,636 (52.0%)	0.026	0.09	1,498 (54.8%)	7,442 (52.5%)	0.046
**U.S. Census Region**									
Midwest; n (%)	7,129 (37.0%)	122 (27.2%)	2,118 (47.6%)	3,998 (36.9%)	**-0.209**	**0.218**	769 (28.1%)	5,916 (41.7%)	**-0.288**
South; n (%)	3,211 (16.7%)	52 (11.6%)	821 (18.4%)	1,992 (18.4%)	**-0.191**	0.000	655 (24.0%)	2,465 (17.4%)	**0.163**
Northeast; n (%)	7,317 (37.9%)	249 (55.6%)	1,248 (28.0%)	3,962 (36.5%)	**0.39**	**-0.183**	1,006 (36.8%)	4,956 (34.9%)	0.04
West; n (%)	1,025 (5.3%)	17 (3.8%)	91 (2.0%)	567 (5.2%)	-0.068	**-0.172**	214 (7.8%)	384 (2.7%)	**0.230**
Missing; n (%)	602 (3.1%)	8 (1.8%)	176 (4.0%)	326 (3.0%)	-0.078	0.054	88 (3.2%)	462 (3.3%)	-0.006
**Insurance Type**									
Uninsured; n (%)	2,655 (13.8%)	70 (15.6%)	441 (9.9%)	1,459 (13.5%)	0.060	**-0.112**	615 (22.5%)	1,643 (11.6%)	**0.293**
MCD Only; n (%)	1,588 (8.2%)	47 (10.5%)	444 (10.0%)	622 (5.7%)	**0.177**	**0.160**	322 (11.8%)	1,004 (7.1%)	**0.161**
MCR Only; n (%)	3,510 (18.2%)	52 (11.6%)	778 (17.5%)	2,335 (21.5%)	**-0.269**	**-0.101**	195 (7.1%)	2,989 (21.1%)	**-0.411**
MCR + MCD; n (%)	688 (3.6%)	19 (4.2%)	219 (4.9%)	361 (3.3%)	0.047	0.081	90 (3.3%)	528 (3.7%)	-0.022
Commercial Only; n (%)	5,841 (30.3%)	175 (39.1%)	1,386 (31.1%)	3,183 (29.3%)	**0.208**	0.039	866 (31.7%)	4,300 (30.3%)	0.030
Commercial+MCR/MCD; n (%)	5,002 (25.9%)	85 (19.0%)	1,186 (26.6%)	2,885 (26.6%)	**-0.182**	0	644 (23.6%)	3,719 (26.2%)	-0.060
**SNF/NH/ALF; n (%)**	1,410 (7.3%)	21 (4.7%)	301 (6.8%)	997 (9.2%)	-0.178	-0.089	64 (2.3%)	1,247 (8.8%)	-0.287
**Overweight or Obese; n (%)**	11,233 (58.3%)	204 (45.5%)	2,874 (64.5%)	6,274 (57.9%)	**-0.250**	**0.136**	1,516 (55.5%)	8,425 (59.4%)	-0.079
**History of smoking; n (%)**	3,838 (19.9%)	41 (9.2%)	900 (20.2%)	2,462 (22.7%)	**-0.375**	-0.061	335 (12.3%)	3,160 (22.3%)	**-0.267**
**Frailty Index; median [IQR]**	0.14 [0.12, 0.18]	0.13 [0.11, 0.16]	0.14 [0.12, 0.18]	0.15 [0.12, 0.19]	**-0.442**	**-0.221**	0.13 [0.11, 0.15]	0.15 [0.12, 0.19]	**-0.442**
**High Risk Conditions‡; median [IQR]**	2.00 [1.00, 3.00]	1.00 [0.00, 2.00]	1.00 [0.00, 2.00]	2.00 [1.00, 3.00]	**-0.368**	**0.130**	1.00 [0.00, 2.00]	1.00 [0.00, 4.00]	**-0.285**
**Charlson Quan median [IQR]**	1.00 [0.00, 3.00]	1.00 [0.00, 3.00]	2.00 [0.00, 4.00]	1.00 [0.00, 3.00]	**-0.100**	0.053	1.00 [0.00, 2.00]	2.00 [1.00, 3.00]	**-0.293**
**Asthma; n (%)**	1,596 (8.3%)	20 (4.5%)	464 (10.4%)	835 (7.7%)	**-0.134**	0.094	236 (8.6%)	1,180 (8.3%)	0.011
**Cancer; n (%)**	1,372 (7.1%)	37 (8.3%)	307 (6.9%)	863 (8.0%)	0.011	-0.042	131 (4.8%)	1,114 (7.9%)	**-0.127**
**Chronic Lung Disease; n (%)**	3,601 (18.7%)	44 (9.8%)	864 (19.4%)	2,249 (20.7%)	**-0.307**	-0.032	348 (12.7%)	2,913 (20.5%)	**-0.211**
**Cardiovascular Disease; n (%)**	10,164 (52.7%)	210 (46.9%)	2,613 (58.7%)	5,965 (55.0%)	**-0.163**	0.075	1,056 (38.7%)	8,061 (56.8%)	**-0.368**
**Diabetes; n (%)**	5,384 (27.9%)	137 (30.6%)	1,474 (33.1%)	2,847 (26.3%)	0.095	**0.149**	772 (28.3%)	4,039 (28.5%)	-0.004
**Immunosuppressed; n (%)**	3,994 (20.7%)	87 (19.4%)	1,003 (22.5%)	2,373 (21.9%)	-0.062	0.014	417 (15.3%)	3,168 (22.3%)	**-0.18**
**Kidney Disease; n (%)**	3,583 (18.6%)	77 (17.2%)	1,030 (23.1%)	2,004 (18.5%)	-0.034	**0.114**	363 (13.3%)	2,863 (20.2%)	**-0.186**
**Liver Disease; n (%)**	846 (4.4%)	18 (4.0%)	181 (4.1%)	490 (4.5%)	-0.025	-0.02	137 (5.0%)	609 (4.3%)	0.033
**Neuro/cognitive Impairment; n (%)**	1,911 (9.9%)	29 (6.5%)	390 (8.8%)	1,328 (12.2%)	**-0.197**	**-0.111**	116 (4.2%)	1,619 (11.4%)	**-0.271**
**Month of COVID-19 Admission**									
March 2020; n (%)	2,753 (14.3%)	77 (17.2%)	848 (19.0%)	1,346 (12.4%)	**0.135**	**0.182**	199 (7.3%)	2,154 (15.2%)	**-0.252**
April 2020; n (%)	5,487 (28.5%)	159 (35.5%)	1,397 (31.4%)	2,871 (26.5%)	**0.196**	**0.108**	693 (25.4%)	4,036 (28.5%)	-0.07
May 2020; n (%)	3,386 (17.6%)	62 (13.8%)	720 (16.2%)	1,899 (17.5%)	**-0.102**	-0.035	557 (20.4%)	2,372 (16.7%)	0.095
June 2020; n (%)	2,388 (12.4%)	62 (13.8%)	436 (9.8%)	1,385 (12.8%)	0.029	-0.095	506 (18.5%)	1,593 (11.2%)	**0.206**
July 2020; n (%)	3,245 (16.8%)	58 (12.9%)	721 (16.2%)	2,000 (18.4%)	**-0.152**	-0.058	508 (18.6%)	2,455 (17.3%)	0.034
August 2020; n (%)	2,025 (10.5%)	30 (6.7%)	332 (7.5%)	1,344 (12.4%)	**-0.195**	**-0.164**	269 (9.8%)	1,573 (11.1%)	-0.043
**Moderate/Severe Symptoms; n (%)**	12,203 (63.3%)	304 (67.9%)	2,929 (65.8%)	6,753 (62.3%)	**0.118**	0.073	1,717 (62.8%)	9,122 (64.3%)	-0.031
**Critical Symptoms; n (%)**	8,524 (44.2%)	201 (44.9%)	1,993 (44.7%)	4,800 (44.3%)	0.012	0.008	1,095 (40.1%)	6,432 (45.4%)	**-0.107**
**COVID-19 Severity**									
Neither; n (%)	9,186 (47.6%)	209 (46.7%)	2,219 (49.8%)	5,000 (46.1%)	0.012	0.074	1,285 (47.0%)	6,657 (46.9%)	0.002
O2/NIV; n (%)	8,763 (45.4%)	192 (42.9%)	1,962 (44.1%)	5,127 (47.3%)	-0.089	-0.064	1,248 (45.7%)	6,549 (46.2%)	-0.01
IMV; n (%)	1,335 (6.9%)	47 (10.5%)	273 (6.1%)	718 (6.6%)	**0.14**	-0.021	199 (7.3%)	977 (6.9%)	0.016

All SMDs with an absolute value > 0.1 have been bolded.

Abbreviations: ALF (Assisted Living Facility); MCR (Medicare); MCR (Medicaid); NH (Nursing Home); SNF (Skilled Nursing Facility)

* SMDs when stratified by Race are calculated using White Race as the referent group

† SMDs when stratified by Ethnicity are calculated using Not Hispanic/Latino Ethnicity as the referent group

‡ High Risk conditions, as defined by the National Strategy for COVID-19 Response include the following: Asthma, Hypertension, Moderate Obesity, Severe Obesity, Diabetes, and Kidney Disease

We described patients initiating five drugs/drug classes; azithromycin (AZM), dexamethasone (DEX), non-dexamethasone corticosteroids of (Non-DEX CSIs; prednisone, methylprednisolone, and hydrocortisone), hydroxychloroquine (HCQ), and remdesivir (RDV). A 90-day washout was applied in order to identify new users of AZM, HCQ, and RDV at admission; a 90-day all-CSI washout was applied to identify new users of DEX and non-DEX CSIs.

To evaluate differences in treatment patterns by race and ethnicity, we estimated adjusted odds ratios (aOR) and two-sided 95% confidence intervals (CI) for receiving each treatment of interest at admission. Patients with non-missing race were included in models stratifying by race, while patients with non-missing ethnicity were included in models stratifying by ethnicity. All covariates adjusted for in the model were selected a priori because they were hypothesized as potential confounders of the relationship between race or ethnicity and treatment patterns. Pre-specified covariates included age, sex, frailty index, comorbidity score, insurance type, census region, skilled nursing/nursing home/assisted living facility (SNF/NH/ALF) status, overweight/obese status, history of smoking, admitting COVID-19 severity, and month of admission. All models were assessed for overfitting and positivity violations. Since all models had greater than 100 subjects per parameter estimated, there were no concerns for overfitting. Positivity of variables was evaluated by examining contingency tables comparing each covariate to the outcome. Model cell counts fewer than five were not observed, except the model of dexamethasone by race stratified by ethnicity, where a cell count of 4 was observed for patients who were Black/AA and Hispanic/Latino and had the outcome of interest; this model was interpreted with caution.

The Aetion Evidence Platform® (2021), a software for real-world data analysis validated for a range of study designs, was used to build analytic cohorts and conduct descriptive analyses [25]. Subsequent logistic regression models were estimated using R (v4.0.3). This study was approved under exemption by the New England Institutional Review Board (#1-9757-1).

## Results

Among the 19,284 patients included in our study cohort, 448 (2.3%) were Asian, 4,454 (23.1%) were Black/AA, 10,845 (56.2%) were White, and 3,537 (18.3%) were missing race. Hispanic/Latino patients represented 14.2% (N = 2,732) of the population, while 73.5% of patients (N = 14,183) were Not Hispanic/Latino (2,329, or 12.1% had a missing ethnicity). In total, we had 15,745 (81.6%) patients with non-missing race, and 16,915 (87.7%) patients with non-missing ethnicity **(S2 Fig).**

Baseline clinical factors, comorbidities, and severity at admission varied by race and ethnicity. White patients tended to be older than other racial subgroups, with an average age of 60.44 years old (versus 56.56 and 56.12 years old among Asian and Black/AA patients, respectively), had higher frailty scores (median: 0.15 vs. 0.13 and 0.14 among Asian and Black/AA patients, respectively), and were more likely to have neurocognitive impairment (12.2% vs. 6.5% and 8.8% among Asian and Black/AA patients, respectively). Relative to White patients, Black/AA patients were more likely to be overweight or obese (64.5% vs. 57.9%), have diabetes (33.1% vs. 26.3%), and have kidney disease (23.1% vs. 18.5%). Hispanic/Latino patients were younger (50.14 vs. 59.72 years old) and had a lower comorbidity score, relative to Not Hispanic/Latino patients (median: 1 vs. 2).

Full results comparing patients by race, and by ethnicity can be viewed in **Table 1,** with results for patients with missing race or ethnicity included in **S1** and **S2 Tables** for results stratified by both race and ethnicity.

### COVID-19 treatment patterns at admission

**Fig 1:**
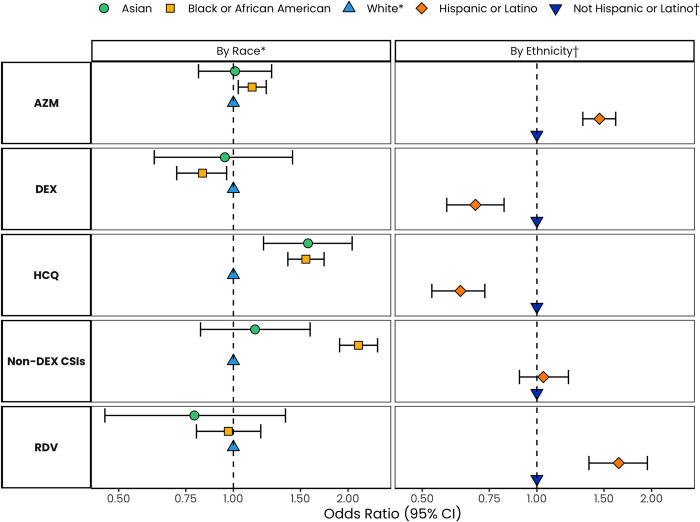
Adjusted odds of receiving COVID-19 treatments at admission by race and by ethnicity. * Regression Models stratified by Race are calculated using White Race as the referent group, and adjust for the following: race, age, sex, frailty, Charlson-Quan score, Medicare insurance, Medicaid insurance, Commercial insurance, COVID-19 severity, region, SNF/NH/ALF, overweight/obesity, smoking, and month of admission. The analytic cohort included 15,745 patients after excluding those with Other/Unknown race. † Regression Models stratified by Ethnicity are calculated using Not Hispanic/Latino Ethnicity as the referent group, and adjust for the following: ethnicity, age, sex, frailty, Charlson-Quan score, Medicare insurance, Medicaid insurance, Commercial insurance, COVID-19 severity, region, SNF/NH/ALF, overweight/obesity, smoking, and month of admission. The analytic cohort included 16,915 patients after excluding those with missing ethnicity.

Overall, the most common treatments of interest received were azithromycin, hydroxychloroquine, and Non-DEX CSIs overall (AZM: 31.5%, HCQ: 18.1%, and Non-DEX CSIs: 11.3%) **(Table 2).** Compared to White patients, the odds of receiving AZM were higher among Black/AA patients, (aOR [95% CI]: 1.12, [1.03, 1.22]), while the odds of receiving HCQ were higher among both Asian patients (1.57, [1.19, 2.05]) and Black/AA patients (1.55 [1.39, 1.73]) after adjustment. Black/AA patients had lower odds of receiving DEX (0.83 [0.71, 0.96]) and higher odds of receiving a Non-DEX CSI (2.13 [1.09, 2.39]) **(Table 3; Fig 1)**. Hispanic/Latino patients had higher odds of receiving AZM and RDV (aOR [95% CI]:1.46 [1.32, 1.61]) and 1.64 [1.37, 1.95], respectively), and lower odds of receiving HCQ and DEX (0.63 [0.53, 0.73]) and 0.69 [0.58, 0.82], respectively), versus Not Hispanic/Latino patients **(Table 3; Fig 1)**.

**Table 2:** COVID-19 treatments received at admission by race, and by ethnicity.

	Overall	Stratified by Race (N = 15,745)			Stratified by Ethnicity (N = 16,915)	
	Total Cohort N = 19,284	Asian N = 448	Black or African American N = 4,454	White N = 10,845	Hispanic/ Latino N = 2,732	Not Hispanic/Latino N = 14,183
**COVID-19 Treatments of Interest at Admission**						
**Azithromycin**	6,069 (31.5%)	156 (34.8%)	1,449 (32.5%)	3,249 (30.0%)	985 (36.1%)	4,416 (31.1%)
**Corticosteroids Other than Dexamethasone** *	2,181 (11.3%)	43 (9.6%)	806 (18.1%)	946 (8.7%)	280 (10.2%)	1,721 (12.1%)
Hydrocortisone	110 (0.6%)	5 (1.1%)	24 (0.5%)	58 (0.5%)	13 (0.5%)	84 (0.6%)
Methylprednisolone	1,840 (9.5%)	29 (6.5%)	647 (14.5%)	850 (7.8%)	236 (8.6%)	1,464 (10.3%)
Prednisone	988 (5.1%)	19 (4.2%)	423 (9.5%)	368 (3.4%)	133 (4.9%)	781 (5.5%)
**Dexamethasone**	1,731 (9.0%)	31 (6.9%)	296 (6.6%)	1130 (10.4%)	215 (7.9%)	1,343 (9.5%)
**Hydroxychloroquine**	3,482 (18.1%)	120 (26.8%)	1,100 (24.7%)	1,678 (15.5%)	294 (10.8%)	2,745 (19.4%)
**Remdesivir**	1,054 (5.5%)	18 (4.0%)	175 (3.9%)	628 (5.8%)	248 (9.1%)	729 (5.1%)

*Individual Non-DEX CSIs may not be mutually exclusive (i.e., a patient may have more than one CSI administered at admission) and may sum to more than the combined Non-DEX CSI total.

**Table 3:** Unadjusted and adjusted odds of receiving COVID-19 treatments at admission by race, ethnicity, and race stratified by ethnicity.

	Primary Analysis*										Secondary Analysis†											
	By Race						By Ethnicity				By Race Stratified by Ethnicity											
	Asian		Black/AA		White		Hispanic/Latino		Not Hispanic /Latino		Black/AA				Other/Unknown Race				White			
											Hispanic/Latino		Not Hispanic/Latino		Hispanic/Latino		Not Hispanic/Latino		Hispanic/Latino		Not Hispanic/Latino	
	OR	(95% CI)	OR	(95% CI)	OR	(95% CI)	OR	95% CI	OR	(95% CI)	OR	(95% CI)	OR	(95% CI)	OR	(95% CI)	OR	(95% CI)	OR	(95% CI)	OR	(95% CI)
**Azithromycin**																						
Unadjusted Model	**1.25**	**(1.02, 1.52)**	**1.13**	**(1.05, 1.22)**	ref		**1.25**	**(1.14, 1.36)**	ref		1.21	(0.81, 1.81)	**1.15**	**(1.06, 1.24)**	**1.44**	**(1.29, 1.61)**	**1.25**	**(1.07, 1.46)**	**1.18**	**(1.03, 1.35)**	ref	
Full Model	1.01	(0.81, 1.26)	**1.12**	**(1.03, 1.22)**	ref		**1.46**	**(1.32, 1.61)**	ref		1.13	(0.73, 1.77)	**1.16**	**(1.06, 1.27)**	**1.6**	**(1.41, 1.82)**	**1.20**	**(1.01, 1.43)**	**1.53**	**(1.31, 1.78)**	ref	
**Corticosteroids Other than Dexamethasone**																						
Unadjusted Model	1.11	(0.81, 1.53)	**2.31**	**(2.09, 2.56)**	ref		**0.83**	**(0.72, 0.94)**	ref		**1.88**	**(1.11, 3.17)**	**2.23**	**(2.00, 2.48)**	**1.48**	**(1.26, 1.74)**	**1.51**	**(1.21, 1.88)**	**0.55**	**(0.42, 0.73)**	ref	
Full Model	1.14	(0.82, 1.59)	**2.13**	**(1.90, 2.39)**	ref		1.04	(0.90, 1.21)	ref		**1.99**	**(1.15, 3.43)**	**2.17**	**(1.93, 2.45)**	**1.81**	**(1.52, 2.17)**	**1.60**	**(1.27, 2.03)**	0.78	(0.58, 1.05)	ref	
**Dexamethasone**																						
Unadjusted Model	**0.64**	**(0.44, 0.93)**	**0.61**	**(0.54, 0.70)**	ref		**0.82**	**(0.70, 0.95)**	ref		**0.32**	**(0.12, 0.86)**	**0.57**	**(0.49, 0.66)**	0.84	(0.70, 1.00)	**0.69**	**(0.53, 0.91)**	**0.49**	**(0.37, 0.64)**	ref	
Full Model	0.95	(0.62, 1.43)	**0.83**	**(0.71, 0.96)**	ref		**0.69**	**(0.58, 0.82)**	ref		0.33	(0.10, 1.10)	**0.74**	**(0.63, 0.88)**	0.88	(0.71, 1.10)	0.88	(0.65, 1.19)	**0.36**	**(0.27, 0.49)**	ref	
**Hydroxychloroquine**																						
Unadjusted Model	**2.00**	**(1.61, 2.48)**	**1.79**	**(1.64, 1.95)**	ref		**0.50**	**(0.44, 0.57)**	ref		1.54	(0.97, 2.43)	**1.78**	**(1.62, 1.95)**	**0.64**	**(0.54, 0.76)**	**1.52**	**(1.27, 1.81)**	**0.54**	**(0.43, 0.67)**	ref	
Full Model	**1.57**	**(1.20, 2.05)**	**1.55**	**(1.39, 1.73)**	ref		**0.63**	**(0.53, 0.73)**	ref		1.43	(0.81, 2.53)	**1.59**	**(1.42, 1.79)**	**0.67**	**(0.55, 0.83)**	**1.48**	**(1.18, 1.86)**	0.86	(0.66, 1.12)	ref	
**Remdesivir**																						
Unadjusted Model	0.68	(0.42, 1.10)	**0.67**	**(0.56, 0.79)**	ref		**1.84**	**(1.59, 2.14)**	ref		1.70	(0.88, 3.28)	0.65	(0.54, 0.78)	**1.77**	**(1.47, 2.14)**	0.93	(0.67, 1.28)	**1.41**	**(1.10, 1.79)**	ref	
Full Model	0.79	(0.46, 1.37)	0.97	(0.80, 1.18)	ref		**1.64**	**(1.37, 1.95)**	ref		**2.21**	**(1.01, 4.86)**	0.97	(0.79, 1.19)	**1.97**	**(1.57, 2.46)**	1.22	(0.85, 1.75)	1.23	(0.93, 1.62)	ref	

* Primary Analysis uses White race as the referent group by race and Not Hispanic/Latino as the referent group by ethnicity.

† Secondary Analysis uses Not Hispanic/Latino Ethnicity and White race as the referent group.

In our secondary analyses, Black/AA patients with either Hispanic/Latino or Not Hispanic/Latino ethnicity had higher odds of receiving Non-DEX CSI (aOR [95% CI]: 1.99 [1.15, 3.43]; 2.10 [1.93, 2.4]) relative to White, Not Hispanic/Latino patients **(Table 3).** Black/AA patients with Not Hispanic/Latino ethnicity also had higher odds of receiving AZM (aOR [95% CI: 1.16 [1.06, 1.27]) and HCQ (aOR [95% CI: 1.59 [1.42, 1.79]), relative to White, Not Hispanic Latino patients. White patients with Hispanic/Latino ethnicity had higher odds of receiving AZM (1.53 [1.31, 1.78]) and lower odds of receiving DEX (0.36 [0.27, 0.49]) relative to Not Hispanic/Latino **(Table 3)**. Models for DEX should be interpreted with caution, due to a low cell count (N = 4) of patients who were Black/AA and Hispanic/Latino and had the outcome of interest.

## Discussion

Similar to other recently published studies, our study identified differences in baseline demographic and clinical characteristics of patients by race and ethnicity [9, 10]. In particular, we found that White patients were older and more frail, while Black/AA patients had slightly more *high risk conditions* (e.g., diabetes, kidney disease, obesity), relative to other racial subgroups. We also identified differences in treatments received at hospital admission by race and ethnicity after adjusting for differences in baseline characteristics.

Learnings about the safety and effectiveness of the treatments for COVID-19 evolved in the early months of the pandemic. After receiving an Emergency Use Authorization (EUA) in April 2020, several studies demonstrated lack of clinical benefit for HCQ, and the EUA was ultimately revoked. Full results of the RECOVERY trial, along with other more recent studies, have shown HCQ to be ineffective at best and harmful at worst [26–28]. AZM was not shown to improve mortality outcomes in either the RECOVERY trial or in other observational analyses [12, 29]. Our study found that some racial and ethnic minority groups were more likely to receive AZM and HCQ, suggesting that historically marginalized patient populations were more likely to receive treatments with limited effectiveness or safety concerns in an inpatient setting in the early months of the pandemic.

Our study found that White patients may have been more likely to have received DEX than racial and ethnic minority patients. DEX and other corticosteroids have been shown to lower 28-day mortality among some hospitalized patients. [30, 31]. These findings were widely publicized in both the medical literature and lay press and analyses in HealthVerity data showed that use of DEX sharply increased after their release [32, 33].

Our findings build upon existing literature on COVID-19 health disparities by evaluating treatment patterns by both race and ethnicity, which is not complete in many RWD sources. A recently published parallel analysis of HCQ and AZM in hospitalized patients reported higher use among Black/AA patients relative to White patients in three of the five datasets analyzed [12]. Another study reported lower rates of DEX, MPRED, and RDV, and higher rates of HCQ among Black/AA patients relative to White patients [13]. However, these studies did not stratify by or control for other baseline differences. Our findings indicate that racial and ethnic differences in COVID-19 treatment for hospitalized patients remained after controlling for covariates such as age, comorbidities, and COVID-19 severity at admission, suggesting racial and ethnic disparities in COVID-19 treatments in the inpatient setting. These disparities may help to explain the higher COVID-19 mortality observed among minority groups as use of treatments with limited evidence of effectiveness or with safety concerns may lead to increased morbidity and mortality among already high-risk populations [4–8]. Reasons for the disparities in treatment patterns remain unclear, though a recent study has suggested structural disparities at the hospital or community-level [34].

While our study has several strengths, including a large cohort of hospitalized patients, with a low degree of missingness for race and ethnicity and adjustment for several key confounders, there are limitations. First, our data were derived from EHRs and claims, which have limitations in quality of race and ethnicity data. For example, our research was limited to the categories for race and ethnicity available in the data, which do not distinguish American Indian/Alaska Native or Native Hawaiian/Pacific Islander race. These patients were possibly misclassified or grouped in the “Other/Unknown” category. Furthermore, the mutually exclusive categorization did not record more than one race per patient, so multiracial patients may either have had only one of their race values captured or may have been grouped with “Other/Unknown”. We were also not able to differentiate between patients with race recorded as “Other”, a value not accepted in the OMB standards, and patients with a missing race value. Second, the data included few Asian patients limiting our ability to identify disparities associated with Asian race. Third, our study population (derived from Optum EHR data), which mostly comprised commercially insured individuals, may not be representative of the broader U.S. population or particular groups such as those age 65 years or greater. Fourth, while our multivariable models included month of admission and region, we did not include more granular categorizations of time or geography, which may impact racial and ethnic disparities given the rapid and varied evolution of the COVID-19 pandemic in different geographies. Fifth, our decision to limit the study population to a cohort that interacted with the health care system in the last six months may have excluded the most vulnerable patients with limited healthcare access. ​​Sixth, while hospital type (academic, non-academic, or community-based) was considered due to its potential association with treatment choices, this variable did not have complete capture in the data for inclusion [34]. Lastly, our study is also impacted by limitations of EHR datasets more broadly; EHR data relies on the accurate recording of patients’ interactions with the health system and capture of demographic information, including race and ethnicity, by healthcare providers. EHR records do not allow us to distinguish between race and ethnicity data reported by physicians versus data reported by patients. Data collection and aggregation may be differential by race or ethnicity (e.g., some insurance types may be overrepresented, EHR fields could be differentially missing). Real-world data including EHR like our data source often lacks additional indicators of socioeconomic status, such as economic or educational status, factors that may modify the relationship between race or ethnicity and treatment patterns.

The COVID-19 pandemic is just one of many examples of how a patient’s race and ethnicity may influence the exposure to risk, access to care, patterns of care, and ultimately, the outcomes they experience. While recent perspective pieces have focused on the need for diversity in clinical trials to support more inclusive research and the advancement towards health equity, increased diversity and inclusion is needed across all types of research. Highlighting and addressing the current limitations on race and ethnicity data through optimizing real-world data sources, such as EHR, will allow us to include diverse populations and align with recent perspectives advocating for increasing diversity in clinical trials and other evidence bases [35].

## Conclusion

Similar to other recently published studies, our study identified differences in demographic and clinical characteristics of patients by race and ethnicity [9, 10]. However, our findings demonstrated that these differences could not explain differential treatment patterns by race and ethnicity. These data suggest potential systematic racial and ethnic disparities in COVID-19 treatment in the inpatient setting occurring at a period of time characterized by rapid evolution of clinical knowledge at patterns of care [4–8]. Although our study period focused on the early months of the pandemic and the treatment and prevention landscape of COVID-19 continues to evolve, the emergence of novel variants of COVID-19 (e.g., delta and omicron) introduce new periods of uncertainty and acceleration of scientific development reminiscent of the early months of the pandemic in the United States. Therefore, lessons learned from our study period can not only help inform policies and strategies designed to mitigate disparities that may be associated with the treatment of new variants, but also other times marked by tumultuous changes in the clinical landscape and patient care.

More complete capture of race and ethnicity across other data types and sources is needed to better understand these disparities; additional studies should investigate potential explanatory factors for differential treatment patterns by race and ethnicity, and how these difference impact disparities in outcomes in the COVID-19 pandemic and future therapeutic areas characterized by accelerated evolution of the treatment landscape.

## Supporting information

S1 Fig Study diagram. (TIF)

S2 Fig CONSORT table. (TIF)

S3 Fig Optum COVID-19 data schema and summary of patient counts and event-specific data ranges. (TIF)

S1 Table Baseline and admitting characteristics by race, and by ethnicity (full results). (DOCX)

S2 Table Baseline and admitting characteristics by race stratified by ethnicity (aim 1 + 2, secondary analysis). (DOCX)

S3 Table Optum COVID-19 de-identified electronic health records. The Optum COVID-19 de-identified electronic health records (EHR) is sourced from laboratories and hospital and emergency department EHRs from integrated delivery networks (IDNs) and smaller outpatient clinics from all over the country. The data in the analysis is entirely inpatient and includes diagnosis data, laboratory data with results, procedures, vital sign measurements, prescriptions written, and medications administered. Sourced from the legacy Humedica database, now Optum EHR, the limited dataset includes a subset of patients as described in the COVID-specific data selection criteria. Data capture began February 1, 2020 and ended on September 24, 2020 with no scheduled updates and includes approximately 2 million patients (N = 2,018,728). If patients were already in the Optum EHR database, patient history was included. The underlying data is representative of the US, but the COVID-19 cut of data is skewed towards the Midwest and Northeast.
(DOCX)
