# Supplementary figures and images for: Describing characteristics and treatment patterns of patients hospitalized with COVID-19 by race and ethnicity in a national RWD during the early months of the pandemic
Source: PLoS One. 2022 Sep 26;17(9):e0267815. doi: 10.1371/journal.pone.0267815 (PMC9512177; doi:10.1371/journal.pone.0267815)

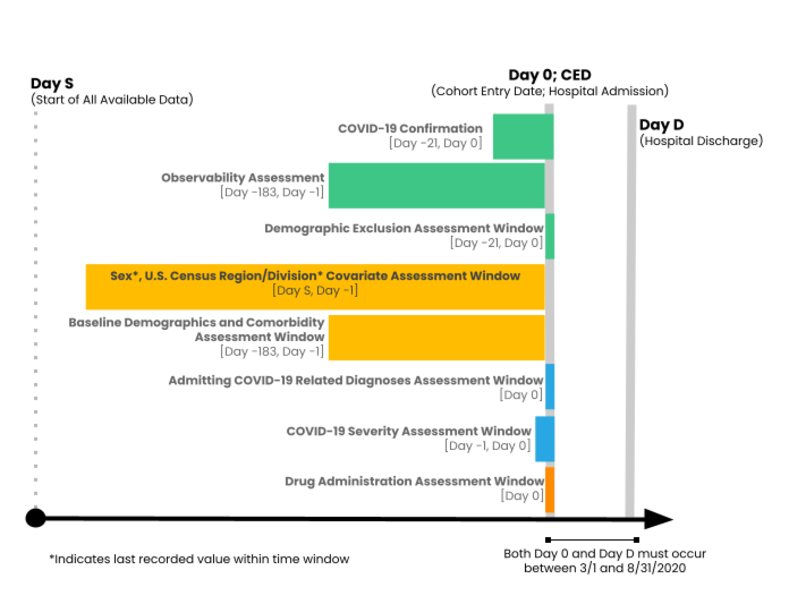

Supplement: S1 Fig — (TIF) [file pone.0267815.s001.tif]

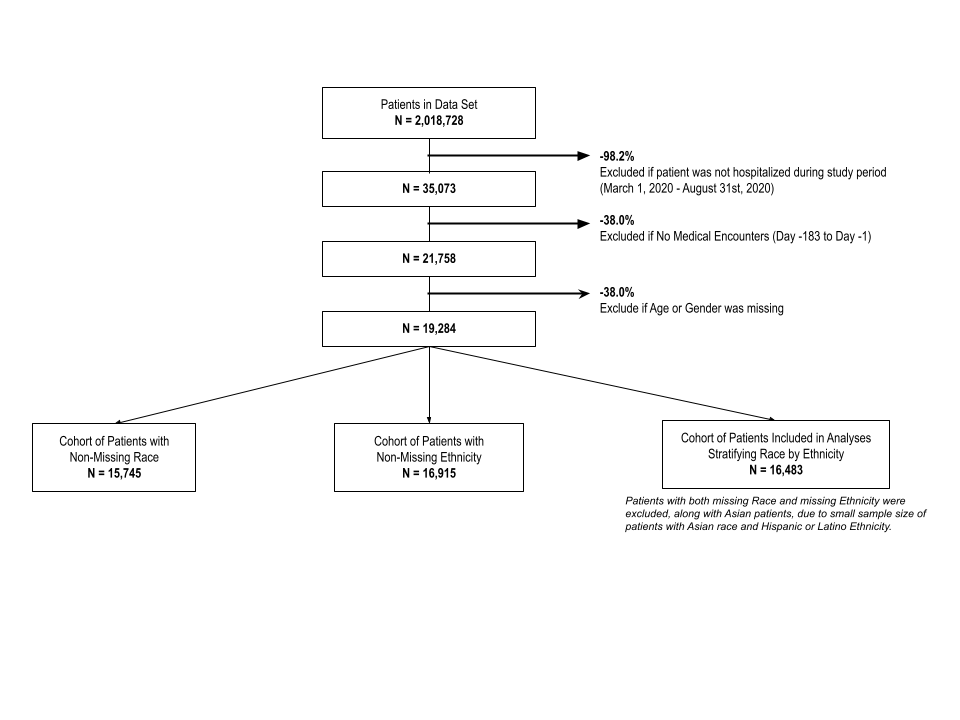

Supplement: S2 Fig — (TIF) [file pone.0267815.s002.tif]

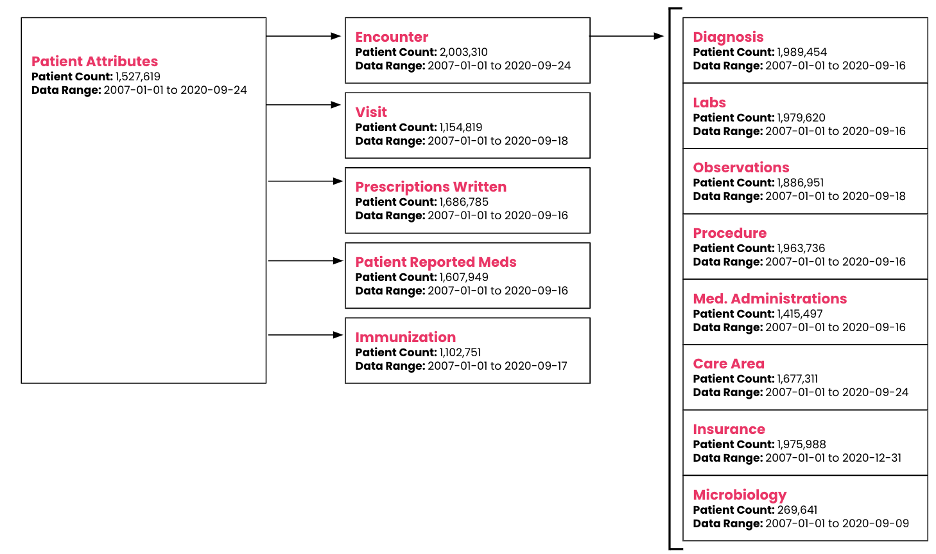

Supplement: S3 Fig — (TIF) [file pone.0267815.s003.tif]
