# Supplementary material for: Describing characteristics and treatment patterns of patients hospitalized with COVID-19 by race and ethnicity in a national RWD during the early months of the pandemic
Source: PLoS One. 2022 Sep 26;17(9):e0267815. doi: 10.1371/journal.pone.0267815 (PMC9512177; doi:10.1371/journal.pone.0267815)
Supplement: S1 Table — (DOCX) [file pone.0267815.s004.docx]

**S1 Table**: Baseline and Admitting Characteristics by Race, and by Ethnicity (Full Results)

|  | Overall | Stratified by Race | | | | | | | Stratified by Ethnicity | | | | |
| --- | --- | --- | --- | --- | --- | --- | --- | --- | --- | --- | --- | --- | --- |
|  | **Total Cohort**  N = 19,284 | **Asian**  N = 448 | **Black/AA**  N = 4,454 | **White**  N = 10,845 | **Other/Missing**  N = 3,537 | **Asian**  (SMD*) | **Black/AA**  (SMD*) | **Other/Missing**  (SMD*) | **Hispanic/**  **Latino**  N = 2,732 | **Not Hispanic/**  **Latino**  N = 14,183 | **Unknown**  N = 2,329 | **Hispanic**  **/Latino**  (SMD†) | **Unknown**  (SMD†) |
| **DEMOGRAPHIC CHARACTERISTICS AT BASELINE** | | | | | | | | | | | | | |
| **Age** |  |  |  |  |  |  |  |  |  |  |  |  |  |
| ...mean (sd) | 57.73 (18.23) | 56.56 (17.38) | 56.12 (17.01) | 60.44 (18.07) | 51.58 (18.54) | **-0.219** | **-0.246** | **-0.484** | 50.14 (17.90) | 59.72 (17.66) | 54.55 (19.20) | **-0.539** | **-0.280** |
| ...median [IQR] | 60 [46, 72] | 58 [44, 70] | 58 [45, 69] | 63 [49, 75] | 53 [37, 66] |  |  |  | 51 [37, 63] | 62 [49, 73] | 57 [39, 70] |  |  |
| **Sex** |  |  |  |  |  |  |  |  |  |  |  |  |  |
| ...Female; n (%) | 10,205 (52.9%) | 239 (53.3%) | 2,517 (56.5%) | 5,636 (52.0%) | 1,813 (51.3%) | 0.026 | 0.09 | -0.014 | 1,498 (54.8%) | 7,442 (52.5%) | 1,265 (53.4%) | 0.046 | 0.018 |
| **U.S. Census Region** |  |  |  |  |  |  |  |  |  |  |  |  |  |
| ...Midwest; n (%) | 7,129 (37.0%) | 122 (27.2%) | 2,118 (47.6%) | 3,998 (36.9%) | 891 (25.2%) | **-0.209** | **0.218** | **-0.255** | 769 (28.1%) | 5,916 (41.7%) | 444 (18.7%) | **-0.288** | **-0.517** |
| ...South; n (%) | 3,211 (16.7%) | 52 (11.6%) | 821 (18.4%) | 1,992 (18.4%) | 346 (9.8%) | **-0.191** | 0.000 | **-0.249** | 655 (24.0%) | 2,465 (17.4%) | 91 (3.8%) | **0.163** | **-0.453** |
| ...Northeast; n (%) | 7,317 (37.9%) | 249 (55.6%) | 1,248 (28.0%) | 3,962 (36.5%) | 1,858 (52.5%) | **0.39** | **-0.183** | **0.326** | 1,006 (36.8%) | 4,956 (34.9%) | 1,355 (57.2%) | 0.04 | **0.459** |
| ...West; n (%) | 1,025 (5.3%) | 17 (3.8%) | 91 (2.0%) | 567 (5.2%) | 350 (9.9%) | -0.068 | **-0.172** | **0.179** | 214 (7.8%) | 384 (2.7%) | 427 (18.0%) | **0.230** | **0.519** |
| ...Missing; n (%) | 602 (3.1%) | 8 (1.8%) | 176 (4.0%) | 326 (3.0%) | 92 (2.6%) | -0.078 | 0.054 | -0.024 | 88 (3.2%) | 462 (3.3%) | 52 (2.2%) | -0.006 | -0.067 |
| **Insurance Type** |  |  |  |  |  |  |  |  |  |  |  |  |  |
| ...Uninsured; n (%) | 2,655 (13.8%) | 70 (15.6%) | 441 (9.9%) | 1,459 (13.5%) | 685 (19.4%) | 0.060 | **-0.112** | **0.16** | 615 (22.5%) | 1,643 (11.6%) | 397 (16.8%) | **0.293** | **0.149** |
| ...MCD Only; n (%) | 1,588 (8.2%) | 47 (10.5%) | 444 (10.0%) | 622 (5.7%) | 475 (13.4%) | **0.177** | **0.160** | **0.264** | 322 (11.8%) | 1,004 (7.1%) | 262 (11.1%) | **0.161** | **0.139** |
| ...MCR Only; n (%) | 3,510 (18.2%) | 52 (11.6%) | 778 (17.5%) | 2,335 (21.5%) | 345 (9.8%) | **-0.269** | **-0.101** | **-0.326** | 195 (7.1%) | 2,989 (21.1%) | 326 (13.8%) | **-0.411** | **-0.193** |
| ...MCR + MCD; n (%) | 688 (3.6%) | 19 (4.2%) | 219 (4.9%) | 361 (3.3%) | 89 (2.5%) | 0.047 | 0.081 | -0.048 | 90 (3.3%) | 528 (3.7%) | 70 (3.0%) | -0.022 | -0.039 |
| ...Commercial Only; n (%) | 5,841 (30.3%) | 175 (39.1%) | 1,386 (31.1%) | 3,183 (29.3%) | 1,097 (31.0%) | **0.208** | 0.039 | 0.037 | 866 (31.7%) | 4,300 (30.3%) | 675 (28.5%) | 0.030 | -0.04 |
| ...Commercial+MCR/MCD; n (%) | 5,002 (25.9%) | 85 (19.0%) | 1,186 (26.6%) | 2,885 (26.6%) | 846 (23.9%) | **-0.182** | 0 | -0.062 | 644 (23.6%) | 3,719 (26.2%) | 639 (27.0%) | -0.060 | 0.018 |
| **SNF/NH/ALF; n (%)** | 1,410 (7.3%) | 21 (4.7%) | 301 (6.8%) | 997 (9.2%) | 91 (2.6%) | -0.178 | -0.089 | -0.283 | 64 (2.3%) | 1,247 (8.8%) | 99 (4.2%) | -0.287 | -0.187 |
| **COMORBIDITIES AND RISK FACTORS AT BASELINE** | | | | | | | | | | | | | |
| **Overweight or Obese; n (%)** | 11,233 (58.3%) | 204 (45.5%) | 2,874 (64.5%) | 6,274 (57.9%) | 1,881 (53.2%) | **-0.250** | **0.136** | -0.095 | 1,516 (55.5%) | 8,425 (59.4%) | 1,292 (54.5%) | -0.079 | -0.099 |
| **History of smoking; n (%)** | 3,838 (19.9%) | 41 (9.2%) | 900 (20.2%) | 2,462 (22.7%) | 435 (12.3%) | **-0.375** | -0.061 | **-0.276** | 335 (12.3%) | 3,160 (22.3%) | 343 (14.5%) | **-0.267** | **-0.202** |
| **Frailty Index; median [IQR]** | 0.14 [0.12, 0.18] | 0.13 [0.11, 0.16] | 0.14 [0.12, 0.18] | 0.15 [0.12, 0.19] | 0.13 [0.11, 0.16] | **-0.442** | **-0.221** | **-0.442** | 0.13 [0.11, 0.15] | 0.15 [0.12, 0.19] | 0.13 [0.11, 0.17] | **-0.442** | **-0.442** |
| **High Risk Conditions‡; median [IQR]** | 2.00 [1.00, 3.00] | 1.00 [0.00, 2.00] | 1.00 [0.00, 2.00] | 2.00 [1.00, 3.00] | 1.00 [0.00, 2.00] | **-0.368** | **0.130** | **-0.225** | 1.00 [0.00, 2.00] | 1.00 [0.00, 4.00] | 1.00 [0.00, 3.00] | **-0.285** | **-0.193** |
| **Charlson Quan median [IQR]** | 1.00 [0.00, 3.00] | 1.00 [0.00, 3.00] | 2.00 [0.00, 4.00] | 1.00 [0.00, 3.00] | 1.00 [0.00, 3.00] | **-0.100** | 0.053 | **-0.209** | 1.00 [0.00, 2.00] | 2.00 [1.00, 3.00] | 1.00 [0.00, 2.00] | **-0.293** | **-0.177** |
| **Asthma; n (%)** | 1,596 (8.3%) | 20 (4.5%) | 464 (10.4%) | 835 (7.7%) | 277 (7.8%) | **-0.134** | 0.094 | 0.004 | 236 (8.6%) | 1,180 (8.3%) | 180 (7.6%) | 0.011 | -0.026 |
| **Cancer; n (%)** | 1,372 (7.1%) | 37 (8.3%) | 307 (6.9%) | 863 (8.0%) | 165 (4.7%) | 0.011 | -0.042 | **-0.136** | 131 (4.8%) | 1,114 (7.9%) | 127 (5.4%) | **-0.127** | **-0.100** |
| **Chronic Lung Disease; n (%)** | 3,601 (18.7%) | 44 (9.8%) | 864 (19.4%) | 2,249 (20.7%) | 444 (12.6%) | **-0.307** | -0.032 | **-0.219** | 348 (12.7%) | 2,913 (20.5%) | 340 (14.4%) | **-0.211** | **-0.161** |
| **Cardiovascular Disease; n (%)** | 10,164 (52.7%) | 210 (46.9%) | 2,613 (58.7%) | 5,965 (55.0%) | 1,376 (38.9%) | **-0.163** | 0.075 | **-0.327** | 1,056 (38.7%) | 8,061 (56.8%) | 1,047 (44.2%) | **-0.368** | **-0.254** |
| **Diabetes; n (%)** | 5,384 (27.9%) | 137 (30.6%) | 1,474 (33.1%) | 2,847 (26.3%) | 926 (26.2%) | 0.095 | **0.149** | -0.002 | 772 (28.3%) | 4,039 (28.5%) | 573 (24.2%) | -0.004 | -0.098 |
| **Immunosuppressed; n (%)** | 3,994 (20.7%) | 87 (19.4%) | 1,003 (22.5%) | 2,373 (21.9%) | 531 (15.0%) | -0.062 | 0.014 | **-0.179** | 417 (15.3%) | 3,168 (22.3%) | 409 (17.3%) | **-0.18** | **-0.126** |
| **Kidney Disease; n (%)** | 3,583 (18.6%) | 77 (17.2%) | 1,030 (23.1%) | 2,004 (18.5%) | 472 (13.3%) | -0.034 | **0.114** | **-0.143** | 363 (13.3%) | 2,863 (20.2%) | 357 (15.1%) | **-0.186** | **-0.134** |
| **Liver Disease; n (%)** | 846 (4.4%) | 18 (4.0%) | 181 (4.1%) | 490 (4.5%) | 157 (4.4%) | -0.025 | -0.02 | -0.005 | 137 (5.0%) | 609 (4.3%) | 100 (4.2%) | 0.033 | -0.005 |
| **Neuro/cognitive Impairment; n (%)** | 1,911 (9.9%) | 29 (6.5%) | 390 (8.8%) | 1,328 (12.2%) | 164 (4.6%) | **-0.197** | **-0.111** | **-0.277** | 116 (4.2%) | 1,619 (11.4%) | 176 (7.4%) | **-0.271** | **-0.137** |
| **MONTH OF ADMISSION** | | | | | | | | | | | | | |
| ...March 2020; n (%) | 2,753 (14.3%) | 77 (17.2%) | 848 (19.0%) | 1,346 (12.4%) | 482 (13.6%) | **0.135** | **0.182** | 0.036 | 199 (7.3%) | 2,154 (15.2%) | 400 (16.9%) | **-0.252** | 0.046 |
| ...April 2020; n (%) | 5,487 (28.5%) | 159 (35.5%) | 1,397 (31.4%) | 2,871 (26.5%) | 1,060 (30.0%) | **0.196** | **0.108** | 0.078 | 693 (25.4%) | 4,036 (28.5%) | 758 (32.0%) | -0.07 | 0.076 |
| ...May 2020; n (%) | 3,386 (17.6%) | 62 (13.8%) | 720 (16.2%) | 1,899 (17.5%) | 705 (19.9%) | **-0.102** | -0.035 | 0.062 | 557 (20.4%) | 2,372 (16.7%) | 457 (19.3%) | 0.095 | 0.068 |
| ...June 2020; n (%) | 2,388 (12.4%) | 62 (13.8%) | 436 (9.8%) | 1,385 (12.8%) | 505 (14.3%) | 0.029 | -0.095 | 0.044 | 506 (18.5%) | 1,593 (11.2%) | 289 (12.2%) | **0.206** | 0.031 |
| ...July 2020; n (%) | 3,245 (16.8%) | 58 (12.9%) | 721 (16.2%) | 2,000 (18.4%) | 466 (13.2%) | **-0.152** | -0.058 | **-0.143** | 508 (18.6%) | 2,455 (17.3%) | 282 (11.9%) | 0.034 | **-0.153** |
| ...August 2020; n (%) | 2,025 (10.5%) | 30 (6.7%) | 332 (7.5%) | 1,344 (12.4%) | 319 (9.0%) | **-0.195** | **-0.164** | **-0.11** | 269 (9.8%) | 1,573 (11.1%) | 183 (7.7%) | -0.043 | **-0.117** |
| **COVID-19 SYMPTOMS AND SEVERITY AT ADMISSION** | | | | | | | | | | | | | |
| **Moderate/Severe Symptoms; n (%)** | 12,203 (63.3%) | 304 (67.9%) | 2,929 (65.8%) | 6,753 (62.3%) | 2,217 (62.7%) | **0.118** | 0.073 | 0.008 | 1,717 (62.8%) | 9,122 (64.3%) | 1,364 (57.6%) | -0.031 | **-0.138** |
| **Critical Symptoms; n (%)** | 8,524 (44.2%) | 201 (44.9%) | 1,993 (44.7%) | 4,800 (44.3%) | 1,530 (43.3%) | 0.012 | 0.008 | -0.02 | 1,095 (40.1%) | 6,432 (45.4%) | 997 (42.1%) | **-0.107** | -0.067 |
| **COVID-19 Severity** |  |  |  |  |  |  |  |  |  |  |  |  |  |
| ...Neither; n (%) | 9,186 (47.6%) | 209 (46.7%) | 2,219 (49.8%) | 5,000 (46.1%) | 1,758 (49.7%) | 0.012 | 0.074 | 0.072 | 1,285 (47.0%) | 6,657 (46.9%) | 1,244 (52.5%) | 0.002 | **0.112** |
| ...O2/NIV; n (%) | 8,763 (45.4%) | 192 (42.9%) | 1,962 (44.1%) | 5,127 (47.3%) | 1,482 (41.9%) | -0.089 | -0.064 | **-0.109** | 1,248 (45.7%) | 6,549 (46.2%) | 966 (40.8%) | -0.01 | **-0.109** |
| ...IMV; n (%) | 1,335 (6.9%) | 47 (10.5%) | 273 (6.1%) | 718 (6.6%) | 297 (8.4%) | **0.14** | -0.021 | 0.068 | 199 (7.3%) | 977 (6.9%) | 159 (6.7%) | 0.016 | -0.008 |
| All SMDs with an absolute value > 0.1 have been bolded.  Abbreviations: ALF (Assisted Living Facility); MCR (Medicare); MCR (Medicaid); NH (Nursing Home); SNF (Skilled Nursing Facility)  * SMDs when stratified by Race are calculated using White Race as the referent group  † SMDs when stratified by Ethnicity are calculated using Not Hispanic/Latino Ethnicity as the referent group  ‡ High Risk conditions, as defined by the National Strategy for COVID-19 Response include the following: Asthma, Hypertension, Moderate Obesity, Severe Obesity, Diabetes, and Kidney Disease | | | | | | | | | | | | | |
