# Supplementary material for: Describing characteristics and treatment patterns of patients hospitalized with COVID-19 by race and ethnicity in a national RWD during the early months of the pandemic
Source: PLoS One. 2022 Sep 26;17(9):e0267815. doi: 10.1371/journal.pone.0267815 (PMC9512177; doi:10.1371/journal.pone.0267815)
Supplement: S2 Table — (DOCX) [file pone.0267815.s005.docx]

**S2 Table:** Baseline and Admitting Characteristics by Race Stratified by Ethnicity

(Aim 1 + 2, Secondary Analysis)

|  | **Asian** | | **Black/AA** | | **White** | | **Other/Missing** | |
| --- | --- | --- | --- | --- | --- | --- | --- | --- |
|  | **Hispanic/Latino**  N = 7 | **Not Hispanic/Latino**  N = 425 | **Hispanic/Latino**  N = 106 | **Not Hispanic/Latino**  N = 4,085 | **Hispanic/Latino**  N = 1,047 | **Not Hispanic/Latino**  N = 8.892 | **Hispanic/Latino**  N = 1,572 | **Not Hispanic/Latino**  N = 781 |
| **DEMOGRAPHIC CHARACTERISTICS** | | | | | | | | |
| **Age** |  |  |  |  |  |  |  |  |
| ...mean (sd) | 56.86 (12.77) | 56.51 (17.48) | 49.70 (18.35) | 56.32 (16.96) | 49.47 (18.34) | 62.04 (17.44) | 50.58 (17.57) | 52.75 (18.76) |
| ...median [IQR] | 58 [51, 68] | 58 [44, 715] | 51 [37, 64] | 58 [46, 69] | 50 [36, 63] | 65 [52, 76] | 51 [37, 63] | 55 [39, 68] |
| **Sex** |  |  |  |  |  |  |  |  |
| ...Female; n (%) | 4 (57.1%) | 229 (53.9%) | 66 (62.3%) | 2,308 (56.5%) | 618 (59.0%) | 4,516 (50.8%) | 810 (51.5%) | 389 (49.8%) |
| **U.S. Census Region** |  |  |  |  |  |  |  |  |
| ...Midwest; n (%) | 4 (57.1%) | 114 (26.8%) | 36 (34.0%) | 1,966 (48.1%) | 295 (28.2%) | 3,535 (39.8%) | 434 (27.6%) | 301 (38.5%) |
| ...South; n (%) | 0 (0.0%) | 49 (11.5%) | 4 (3.8%) | 806 (19.7%) | 448 (42.8%) | 1,504 (16.9%) | 203 (12.9%) | 106 (13.6%) |
| ...Northeast; n (%) | 1 (14.3%) | 240 (56.5%) | 61 (57.5%) | 1,107 (27.1%) | 203 (19.4%) | 3,308 (37.2%) | 741 (47.1%) | 301 (38.5%) |
| ...West; n (%) | 0 (0.0%) | 16 (3.8%) | 0 (0.0%) | 45 (1.1%) | 67 (6.4%) | 273 (3.1%) | 147 (9.4%) | 50 (6.4%) |
| ...Missing; n (%) | 2 (28.6%) | 6 (1.4%) | 5 (4.7%) | 161 (3.9%) | 34 (3.2%) | 272 (3.1%) | 47 (3.0%) | 23 (2.9%) |
| **Insurance Type** |  |  |  |  |  |  |  |  |
| ...Uninsured; n (%) | 2 (28.6%) | 67 (15.8%) | 20 (18.9%) | 391 (9.6%) | 277 (26.5%) | 1,068 (12.0%) | 316 (20.1%) | 117 (15.0%) |
| ...MCD Only; n (%) | 0 (0.0%) | 42 (9.9%) | 14 (13.2%) | 402 (9.8%) | 106 (10.1%) | 441 (5.0%) | 202 (12.8%) | 119 (15.2%) |
| ...MCR Only; n (%) | 0 (0.0%) | 50 (11.8%) | 7 (6.6%) | 738 (18.1%) | 85 (8.1%) | 2,085 (23.4%) | 103 (6.6%) | 116 (14.9%) |
| ...MCR + MCD; n (%) | 0 (0.0%) | 18 (4.2%) | 1 (0.9%) | 212 (5.2%) | 45 (4.3%) | 278 (3.1%) | 44 (2.8%) | 20 (2.6%) |
| ...Commercial Only; n (%) | 5 (71.4%) | 163 (38.4%) | 38 (35.8%) | 1,242 (30.4%) | 270 (25.8%) | 2,657 (29.9%) | 553 (35.2%) | 238 (30.5%) |
| ...Commercial+MCR/MCD; n (%) | 0 (0.0%) | 85 (20.0%) | 26 (24.5%) | 1,100 (26.9%) | 264 (25.2%) | 2,363 (26.6%) | 354 (22.5%) | 171 (21.9%) |
| **SNF/NH/ALF; n (%)** | 0 (0.0%) | 21 (4.9%) | 1 (0.9%) | 290 (7.1%) | 27 (2.6%) | 911 (10.2%) | 36 (2.3%) | 25 (3.2%) |
| **COMORBIDITIES AND RISK FACTORS** | | | | | | | | |
| **Overweight or Obese; n (%**) | 2 (28.6%) | 192 (45.2%) | 64 (60.4%) | 2,671 (65.4%) | 568 (54.3%) | 5,164 (58.1%) | 882 (56.1%) | 398 (51.0%) |
| **History of smoking; n (%)** | 0 (0.0%) | 40 (9.4%) | 18 (17.0%) | 849 (20.8%) | 124 (11.8%) | 2,169 (24.4%) | 193 (12.3%) | 102 (13.1%) |
| **Frailty Index; median [IQR]** | 0.14 [0.12, 0.16] | 0.13 [0.11, 0.16] | 0.13 [0.11, 0.16] | 0.14 [0.12, 0.18] | 0.12 [0.11, 0.15] | 0.15 [0.12, 0.19] | 0.13 [0.11, 0.16] | 0.13 [0.11, 0.17] |
| **High Risk Conditions*; median [IQR]** | 1.00 [1.00, 3.00] | 1.00 [0.00, 2.00] | 2.00 [1.00, 3.00] | 2.00 [1.00, 3.00] | 1.00 [0.00, 2.00] | 2.00 [1.00, 3.00] | 1.00 [1.00, 2.00] | 1.00 [0.00, 2.00] |
| **Charlson Quan; median [IQR]** | 1.00 [0.00, 2.00] | 1.00 [0.00, 3.00] | 1.00 [0.00, 3.00] | 2.00 [0.00, 4.00] | 1.00 [0.00, 2.00] | 2.00 [0.00, 4.00] | 1.00 [0.00, 2.00] | 1.00 [0.00, 3.00] |
| **Asthma; n (%)** | 0 (0.0%) | 19 (4.5%) | 15 (14.2%) | 423 (10.4%) | 87 (8.3%) | 679 (7.6%) | 134 (8.5%) | 59 (7.6%) |
| **Cancer; n (%)** | 0 (0.0%) | 37 (8.7%) | 7 (6.6%) | 290 (7.1%) | 57 (5.4%) | 747 (8.4%) | 67 (4.3%) | 40 (5.1%) |
| **Chronic Pulmonary Disease; n (%)** | 0 (0.0%) | 43 (10.1%) | 20 (18.9%) | 808 (19.8%) | 128 (12.2%) | 1,962 (22.1%) | 200 (12.7%) | 100 (12.8%) |
| **Cardiovascular Disease; n (%)** | 3 (42.9%) | 200 (47.1%) | 39 (36.8%) | 2,445 (59.9%) | 399 (38.1%) | 5,079 (57.1%) | 615 (39.1%) | 337 (43.1%) |
| **Diabetes; n (%)** | 3 (42.9%) | 128 (30.1%) | 24 (22.6%) | 1,374 (33.6%) | 293 (28.0%) | 2,327 (26.2%) | 452 (28.8%) | 210 (26.9%) |
| **Immunosuppressed; n (%)** | 0 (0.0%) | 86 (20.2%) | 17 (16.0%) | 943 (23.1%) | 176 (16.8%) | 2,019 (22.7%) | 224 (14.2%) | 120 (15.4%) |
| **Kidney Disease; n (%)** | 0 (0.0%) | 76 (17.9%) | 14 (13.2%) | 977 (23.9%) | 137 (13.1%) | 1,700 (19.1%) | 212 (13.5%) | 110 (14.1%) |
| **Liver Disease; n (%)** | 0 (0.0%) | 16 (3.8%) | 4 (3.8%) | 172 (4.2%) | 44 (4.2%) | 394 (4.4%) | 89 (5.7%) | 27 (3.5%) |
| **MONTH OF ADMISSION** | | | | | | | | |
| ...March 2020; n (%) | 2 (28.6%) | 75 (17.6%) | 14 (13.2%) | 787 (19.3%) | 56 (5.3%) | 1,153 (13.0%) | 127 (8.1%) | 139 (17.8%) |
| ...April 2020; n (%) | 0 (0.0%) | 155 (36.5%) | 38 (35.8%) | 1,270 (31.1%) | 205 (19.6%) | 2,394 (26.9%) | 450 (28.6%) | 217 (27.8%) |
| ...May 2020; n (%) | 1 (14.3%) | 57 (13.4%) | 22 (20.8%) | 651 (15.9%) | 187 (17.9%) | 1,536 (17.3%) | 347 (22.1%) | 128 (16.4%) |
| ...June 2020; n (%) | 1 (14.3%) | 56 (13.2%) | 16 (15.1%) | 393 (9.6%) | 211 (20.2%) | 1,036 (11.7%) | 278 (17.7%) | 108 (13.8%) |
| ...July 2020; n (%) | 2 (28.6%) | 53 (12.5%) | 10 (9.4%) | 675 (16.5%) | 273 (26.1%) | 1,614 (18.2%) | 223 (14.2%) | 113 (14.5%) |
| ...August 2020; n (%) | 1 (14.3%) | 29 (6.8%) | 6 (5.7%) | 309 (7.6%) | 115 (11.0%) | 1,159 (13.0%) | 147 (9.4%) | 76 (9.7%) |
| **COVID-19 SYMPTOMS AND SEVERITY AT ADMISSION** | | | | | | | | |
| **Moderate/Severe Symptoms; n (%)** | 6 (85.7%) | 291 (68.5%) | 72 (67.9%) | 2,698 (66.0%) | 589 (56.3%) | 5,636 (63.4%) | 1,050 (66.8%) | 497 (63.6%) |
| **Critical Symptoms; n (%)** | 5 (71.4%) | 192 (45.2%) | 50 (47.2%) | 1,823 (44.6%) | 343 (32.8%) | 4,061 (45.7%) | 697 (44.3%) | 356 (45.6%) |
| **COVID-19 Severity** |  |  |  |  |  |  |  |  |
| ...Neither; n (%) | 3 (42.9%) | 194 (45.6%) | 50 (47.2%) | 2,034 (49.8%) | 500 (47.8%) | 4,053 (45.6%) | 732 (46.6%) | 376 (48.1%) |
| ...O2/NIV; n (%) | 4 (57.1%) | 184 (43.3%) | 43 (40.6%) | 1,800 (44.1%) | 495 (47.3%) | 4,232 (47.6%) | 706 (44.9%) | 333 (42.6%) |
| ...IMV; n (%) | 0 (0.0%) | 47 (11.1%) | 13 (12.3%) | 251 (6.1%) | 52 (5.0%) | 607 (6.8%) | 134 (8.5%) | 72 (9.2%) |
| Abbreviations: ALF (Assisted Living Facility); MCR (Medicare); MCR (Medicaid); NH (Nursing Home); SNF (Skilled Nursing Facility)  * High Risk conditions, as defined by the National Strategy for COVID-19 Response include the following: Asthma, Hypertension, Moderate Obesity, Severe Obesity, Diabetes, and Kidney Disease | | | | | | | | |
