# Supplementary material for: Describing characteristics and treatment patterns of patients hospitalized with COVID-19 by race and ethnicity in a national RWD during the early months of the pandemic
Source: PLoS One. 2022 Sep 26;17(9):e0267815. doi: 10.1371/journal.pone.0267815 (PMC9512177; doi:10.1371/journal.pone.0267815)
Supplement: S3 Table — The Optum COVID-19 de-identified electronic health records (EHR) is sourced from laboratories and hospital and emergency department EHRs from integrated delivery networks (IDNs) and smaller outpatient clinics from all over the country. The data in the analysis is entirely inpatient and includes diagnosis data, laboratory data with results, procedures, vital sign measurements, prescriptions written, and medications administered. Sourced from the legacy Humedica database, now Optum EHR, the limited dataset includes a subset of patients as described in the COVID-specific data selection criteria. Data capture began February 1, 2020 and ended on September 24, 2020 with no scheduled updates and includes approximately 2 million patients (N = 2,018,728). If patients were already in the Optum EHR database, patient history was included. The underlying data is representative of the US, but the COVID-19 cut of data is skewed towards the Midwest and Northeast. (DOCX) [file pone.0267815.s006.docx]

**S3 Table:** Optum COVID-19 De-identified Electronic Health Records

The Optum COVID-19 de-identified electronic health records (EHR) is sourced from laboratories and hospital and emergency department EHRs from integrated delivery networks (IDNs) and smaller outpatient clinics from all over the country. The data in the analysis is entirely inpatient and includes diagnosis data, laboratory data with results, procedures, vital sign measurements, prescriptions written, and medications administered. Sourced from the legacy Humedica database, now Optum EHR, the limited dataset includes a subset of patients as described in the COVID-specific data selection criteria. Data capture began February 1, 2020 and ended on September 24, 2020 with no scheduled updates and includes approximately 2 million patients (N=2,018,728). If patients were already in the Optum EHR database, patient history was included. The underlying data is representative of the US, but the COVID-19 cut of data is skewed towards the Midwest and Northeast.

| **Data specification** | **Detail** |
| --- | --- |
| **Data model** | Datasets are merged from several EHRs. To complete the timeliness of this data, Optum may impute information to help with this merge. The data are HIPAA-compliant while preserving data integrity. Optum EHR has a unique to themselves data model. |
| **COVID-specific data selection criteria** | Data for a select set of patients are pulled from the broader Optum EHR dataset and included in the COVID dataset if they are COVID “possible”, according to the following data elements.    **ICD-10 diagnosis codes:**  COVID-19 diagnosis *(B97.29, B34.2, U07.1, U07.2)*; influenza-like illness, including acute bronchitis, lower respiratory infection *(B97.29, J12.89, J20.8, J22*, J40*, J98.8)*; exposure to COVID-19 *(Z20.828)*; cough *(R05.*)*; shortness of breath *(R06.02)*; fever *(R50.9)*; acute respiratory distress syndrome *(J80.*)*; screening for COVID-19 *(Z11.59)*    **CPT/HCPCS:**  SARs-CoV-2 (COVID-19) lab test procedures *(86328, 86769, 87635, G2023, G2024, U0001, U0002, U0003, U0004)*  **LOINC (Logical Observation Identifiers Names and Codes):**  SARs-CoV-2 (COVID-19) lab test orders *(94534-5, 94500-6, 94503-0, 94505-5, 94506-3, 94504-8, 94507-1, 94509-7, 94508-9, 94510-5, 94308-4, 94309-2, 94311-8, 94312-6, 94314-2, 94315-9, 94316-7, 94533-7, 94531-1, 94547-7, 94564-2, 94562-6, 94563-4, 94565-9, 94307-6, 94559-2, 94306-8, 94558-4, 94511-3)*  **Lab Test Name:**  Text search corresponding to SARs-CoV-2 (COVID-19) lab test procedures/orders included in the CPT/HCPCS and LOINC lists above |
| **COVID+ definition** | This dataset allows for definition (“COVID-19 confirmed”) by COVID diagnosis in the EHR, and/or positive and presumptive positive NAAT/PCR and serology tests from the labs table. |
